# Bio-Tribocorrosion of Titanium Dental Implants and Its Toxicological Implications: A Scoping Review

**DOI:** 10.1155/2022/4498613

**Published:** 2022-10-21

**Authors:** Sumit Gaur, Rupali Agnihotri, Sacharia Albin

**Affiliations:** ^1^Department of Pedodontics and Preventive Dentistry, Manipal College of Dental Sciences, Manipal, India; ^2^Manipal Academy of Higher Education (MAHE), Manipal 576104, Karnataka, India; ^3^Department of Periodontology, Manipal College of Dental Sciences, Manipal, India; ^4^Engineering Department, Norfolk State University, Norfolk, VA 23504, USA

## Abstract

Bio-tribocorrosion is a phenomenon that combines the essentials of tribology (friction, wear, and lubrication) and corrosion with microbiological processes. Lately, it has gained attention in implant dentistry because dental implants are exposed to wear, friction, and biofilm formation in the corrosive oral environment. They may degrade upon exposure to various microbial, biochemical, and electrochemical factors in the oral cavity. The mechanical movement of the implant components produces friction and wear that facilitates the release of metal ions, promoting adverse oro-systemic reactions. This review describes the bio-tribocorrosion of the titanium (Ti) dental implants in the oral cavity and its toxicological implications. The original research related to the bio-tribo or tribocorrosion of the dental implants was searched in electronic databases like Medline (Pubmed), Embase, Scopus, and Web of Science. About 34 studies included in the review showed that factors like the type of Ti, oral biofilm, acidic pH, fluorides, and micromovements during mastication promote bio-tribocorrosion of the Ti dental implants. Among the various grades of Ti, grade V, i.e., Ti6Al4V alloy, is most susceptible to tribocorrosion. Oral pathogens like *Streptococcus mutans* and *Porphyromonas gingivalis* produce acids and lipopolysaccharides (LPS) that cause pitting corrosion and degrade the TiO_2_. The low pH and high fluoride concentration in saliva hinder passive film formation and promote metal corrosion. The released metal ions promote inflammatory reactions and bone destruction in the surrounding tissues resulting in peri-implantitis, allergies, and hyper-sensitivity reactions. However, further validation of the role of bio-tribocorrosion on the durability of the Ti dental implants and Ti toxicity is warranted through clinical trials.

## 1. Introduction

Tribocorrosion is the science that studies the relationship of wear, chemical, and electrochemical processes [1]. It includes tribology, the study of the mechanisms of friction, lubrication, and wear of the interacting surfaces in relative motion, and corrosion that involves irreversible degradation of materials due to electrochemical interactions with the surrounding environment [2, 3]. However, in the oral environment, organism-related factors, including the metabolic, immunological, microbiological, and biochemical processes, also impact the dental implant deterioration [3–5]. The tribocorrosion, when simulated under biological conditions, is referred to as bio-tribocorrosion, which is more appropriate when considering the dental implants in the oral cavity [3].

Dental implants are the most acceptable way of replacing missing teeth, and Titanium (Ti) and its alloys are considered the ideal dental implant materials owing to their osseointegration property, high biocompatibility, and excellent mechanical properties [6]. It forms a protective Ti oxide (TiO_2_) layer by the migration of oxygen atoms through the interstitial diffusion mechanism. They occupy the free, octahedral interstitial positions in the hexagonal Ti lattice and the available axial positions [7]. In the presence of high temperatures and an oxygenated environment, Ti oxidation is faster leading to oxide formation, followed by oxygen diffusion into its bulk structure [7]. The high solubility of oxygen in the Ti and its stabilizing effect on the crystalline Ti structure promotes formation of an oxygen-enriched layer. It is highly resistant even to aggressive environments like hydrochloric or sulphuric acid, as it prevents the anodic pickling of the substrate. Its compactness and bonding to the substrate enhances the corrosion resistance. Besides, thick oxide layers have improved tribological properties as the top sublayer of the TiO_2_ inhibits metal ion release and its transformation in vitro, promoting osseointegration and bone adhesion [7]. It is a barrier between the environment and the material [2]. However, in the human body, the extracellular body fluids and blood contain aqueous solutions of certain organic substances, dissolved oxygen, various inorganic anions (Cl^−^, HPO4^2−^ HCO^3−^), and cations (Na^+^, K^+^, Ca^2+^, Mg^2+^) along with the amino acids and proteins that may degrade the TiO_2_ layer [7]. The dental implants are exposed to saliva-containing inorganic salts and organic components in the oral cavity. Various factors like food and diseases influence its composition. For instance, low salivary pH following ingestion of acidic beverages or due to infections promotes the Ti dental implant corrosion [7]. Fluoride exposure breaks the continuity of the oxide film and damages the Ti. The penetration of fluoride ions into the oxide layer reduces its protective properties. There is delamination of the oxide layer. Furthermore, any mechanical motion erodes the layer, leading to direct contact of the implant with the environment, which initiates its degradation and corrosion [7].

It generates micrometer and nanometer-sized metal ions that initiate an inflammatory response in the surrounding tissues [2]. The Ti ions were detected in the epithelial cells and macrophages in the exfoliative cytology studies of the peri-implant tissues [8]. They cause aseptic osteolysis, even at low concentrations, leading to premature loosening of the dental implants and peri-implantitis [3, 9]. In the oral cavity, pathogens like *Streptococcus mutans (S. mutans)* and *Porphyromonas gingivalis (P. gingivalis)* attack the TiO_2_ layer and induce corrosion. They produce lipopolysaccharides (LPS) and acids that lower the pH of the surrounding environment. The two factors act synergistically with the micro-movement of the implant in the bone resulting in bio-tribocorrosion, which eventually degrades the Ti surface [2, 3]. The aim of this review was to elaborate on the bio-tribocorrosion of Ti dental implants and their toxicological implications.

## 2. Methods

### 2.1. Search Strategy

The present review was conducted according to the Preferred Reporting Items for Systematic reviews and Meta-Analyses extension for Scoping Reviews (PRISMA-ScR) guidelines. The research question was about the factors involved in the bio-tribocorrosion of dental implants and their toxic implications. A literature search was conducted in four electronic databases, viz., Medline (PubMed), Scopus, Embase, and Web of Science with the help of a combination of keywords like “tribocorrosion” OR “bio-tribocorrosion” AND “Dental” AND “Implants” OR “Dentistry” for evaluating the factors involved in bio-tribocorrosion of implants. Furthermore, keywords like “Periimplantitis” OR “Hypersensitivity” OR “Allergy” OR “Toxicity” AND “Titanium” AND “Corrosion” were applied to identify the articles related to Ti toxicity in the titles, abstracts, or keywords during the initial search.

### 2.2. Inclusion Criteria

Full-texts of original studies in English evaluating the following were included:

- Factors that cause bio-tribo or tribocorrosion of Ti dental implants
- Toxic implications of Ti ions released due to bio-tribo or tribocorrosion of Ti dental implants

### 2.3. Exclusion Criteria

Following types of research were excluded: recommendations, animal studies, conference proceedings, expert statements, reviews, and nonoriginal papers.

### 2.4. Data Extraction and Collection

The data were extracted by the authors independently and the disagreements were resolved by discussion. The study type, aims, and objectives; dental implant material; factor tested for tribocorrosion; laboratory parameters, surface, and electrochemical characterization methods; and toxic reactions, results, and conclusions were recorded.

## 3. Results and Discussion

**Figure 1:**
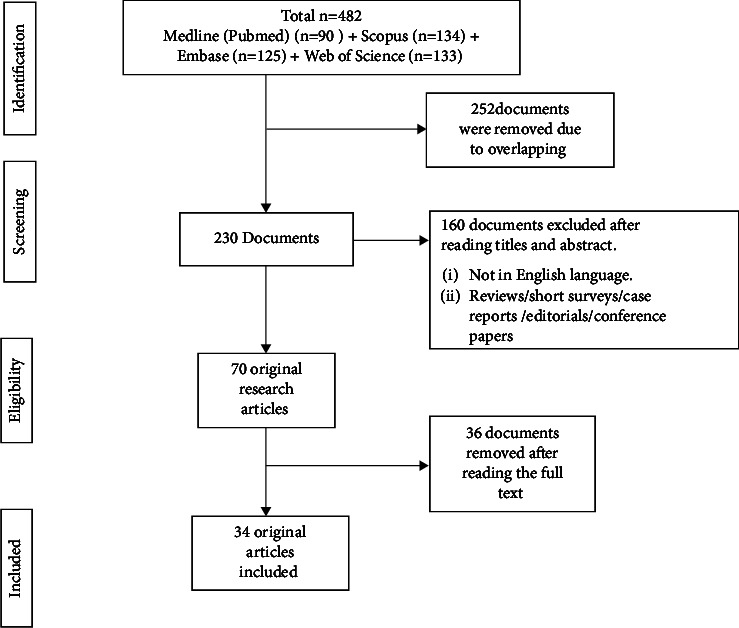
Evidence search for bio-tribocorrosion of dental implants and its toxic implications.

The initial search resulted in 482 articles and after screening the duplicates, the titles and abstracts of 230 articles were read. Finally, full texts of 70 articles were read, of which 34 were included in the review [5, 8–40] (Figure 1). There were 26 in-vitro studies [8–27, 29, 30, 32, 33, 35, 39], 3 ex-vivo studies [5, 28, 38], 3 observational in-vivo studies [31, 34, 36], 1 case-control study [37], and 1 case report [40]. Eighteen studies reported the factors responsible for bio-tribocorrosion [8–25], while 16 reported the toxic effects of the released metal ions [5, 26–40]. The review results are summarized as follows:

### 3.1. Experimental Evaluation of Tribocorrosion of the Ti Dental Implants in Laboratory

**Figure 2:**
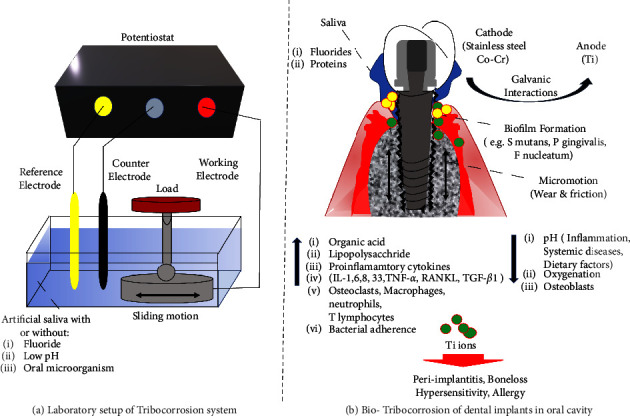
Factors responsible for bio-tribocorrosion of dental implants in oral cavity. (a) Laboratory setup of the tribocorrosion system. (b) Bio-tribocorrosion of dental implants in the oral cavity.

The tribological behaviour of dental implants was assessed using tribometer systems with the help of a pin/ball on a disc [8, 13, 18, 20] or a pin/ball on a plate [9, 11, 12, 16, 19, 22, 24, 25] (Figure 2(a)). Typically, the studies applied loads as low as 20 mN [19] to as high as 20 N [8]. Loads of 1 N [9, 25], 2 N [10, 22, 24], 5 N [13], and 8 N [18, 20] were commonly applied. These forces corresponded to a mean contact pressure ranging from 0.91 MPa [20] to 190 MPa [16] and initial contact pressure of 320 [8, 9, 25] to 998.7 MPa [13, 22, 24].

The tests were performed at a frequency of 1 Hz [8–10, 16, 20–22, 24], 2 Hz [18–20, 25], and 4 Hz [20] during 1000 [16], 1300 [19], 2000 [8], 5000 [10], or 25000 cycles [18]. The strokes ranged from 1 [19, 20] to 2 mm [9, 18, 25]. Tribocorrosion due to fretting [10, 20] and sliding [8, 9, 13, 16, 18, 19, 22, 24, 25] was evaluated. These laboratory parameters simulated the oral environment and masticatory conditions on the implant surfaces.

In reciprocating tests, the configuration of a metallic ball on the ceramic plate is more suitable for in vitro evaluation of the tribological behaviour. When the Ti ball slides against the Zirconia (Zr) plate, the damaged metal surface in contact with the Zr cannot reform the passivating film due to the mechanical action of the Zr and limited oxygen diffusion into the contacting area. Besides, the accumulation of wear debris and material transfer to the Zr surface changes the electrochemical response during testing. It causes screw loosening between the Zr abutment and the Ti implant and mechanical degradation due to micromovements at their direct contact surfaces [19]. The released wear particles become trapped in the contact zone and act as a lubricant or an abrasive component, resulting in the coefficient of friction (CoF) oscillations. As Zr abutments are more rigid, they have greater CoF and more fluctuations than Ti abutments. The loose and rigid wear particles cause mechanical damage and wear-accelerated corrosion [18].

The electrochemical behaviour of the implant material was studied using a three-electrode cell, where the Ti samples were working electrodes, a saturated calomel electrode (SCE) as a reference electrode, and a counter electrode of graphite or platinum [8–11, 13, 15–22, 24, 25]. A potentiostat was used for the electrochemical test. The samples were first immersed in an electrolyte solution under open circuit potential (OCP) for potential stabilization, followed by the potentiodynamic polarization curve estimation. The concentration of anions, composition, and pH of the electrolytic solution are essential determinants for material tribocorrosion performance. In most studies, unstimulated human [19, 21] or artificial saliva based on the Fusayama and Meyer's solution at a pH of 6 was applied to mimic the oral conditions [8–11, 13, 15–18, 20, 22, 24, 25]. Some studies used various concentrations of fluorides and pH to evaluate their influence on tribocorrosion. They are described in a later section.

The studies applied electrochemical methods like OCP [8–10, 13, 16, 18–20, 22–25] and electrochemical impedance spectroscopy [8, 12, 15, 20] to evaluate the corrosion resistance of dental implant materials. The wear loss was measured by profilometry and laser scanning measurements [10]. The material deterioration and wear were studied with the help of scanning electron microscopy (SEM), optical microscope, and 3D interferometry microscopy [8–11, 13, 15–25]. The chemical analyses were done with energy dispersive spectroscopy (EDS) [16, 17, 21].

At steady state, the OCP increased with time due to the formation of the protective passivating layer [8–10, 13, 16, 18–20, 22–25]. After sliding tests, it rapidly decayed due to mechanical removal of the passivating film [8–10, 13, 16, 18–20, 22–25]. A negative OCP in the presence of low pH and high fluoride concentration showed failure of re-passivation with rapid surface degradation [9, 23].

**Figure 3:**
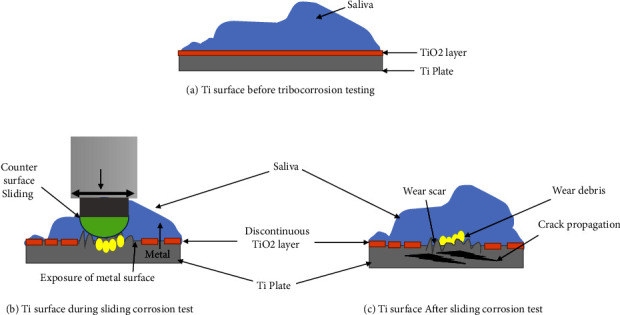
Structural changes in Ti surface following Tribocorrosion testing. (a) Ti surface before tribocorrosion testing. (b) Ti surface during sliding corrosion test. (c) Ti surface after sliding corrosion test.

Surface analysis with different techniques showed rough and deeper wear tracks following sliding tests, indicative of wear loss [8–10, 13, 18–22, 24]. The SEM observation of Ti6Al4V alloy typically showed hexagonal grains. In contrast, atomic force microscopy and white light interferometry images showed a smooth morphology owing to the finely polished surface before tribocorrosion testing [8, 19] (Figure 3(a)).

Following the tribocorrosion test, a wear scar with well-defined boundaries was observed on the Ti dental implants (Figure 3(b)). The SEM images of the wear scars revealed a worn surface characterized by wear marks aligned in a sliding direction. The boundary of the wear scar exhibited some smearing due to the spreading of the wear debris (Figure 3(c)) [8, 9, 11]. Sliding removed the passive protective film and exposed the Ti to active corrosion. Inside the wear scar, there was severe material damage with the accumulation of the wear debris. The debris particles in the center of the wear scar induce oscillations [8, 9]. The cracking and delamination of these particles are accelerated by hardening due to oxidation [9, 11].

Various factors like concentration of LPS, fluorides, type of surfaces in contact, and fretting frequencies during sliding influence the wear-accelerated corrosion and surface roughness. The concentration of LPS was more inside the wear scar leading to increased delamination, cracking with fatigue, and weight loss (Figure 3(c)). The mechanical wear during sliding was related to the metal detached from the surface. Before sliding, the polysaccharide part of LPS attacked the oxide film and induced some defects. The exposed Ti surface was more susceptible to corrosion. After removing the remaining passive film during sliding, ions were exchanged between Ti metal and the saliva. Some debris were formed, and a new Ti surface was attacked by LPS leading to increased total wear loss. Although a new passive film was formed when the sliding stopped, it was less protective than the native film [8]. Increased delaminated areas with cracks perpendicular to the sliding direction were also observed on wear scars on commercially pure titanium (CpTi) rubbed in artificial saliva with 1000 ppm fluoride [9, 13, 22].

The SEM images of the groups where two Ti surfaces were in contact showed characteristic wear patterns with rows of light grooving. In contrast, Zr showed scales that could delaminate and promote mechanical damage and wear-accelerated corrosion. The penetration of the harder material into the metal formed a wear track susceptible to corrosion [18]. Similarly, greater plastic deformation and hardness of Ti13Nb3Zr alloy compared to CpTi4 also increased the abrasive wear of pure Ti [9]. It was observed that human saliva minimized the sliding contact between the body and counter body and reduced friction and wear. It significantly reduced the adhesion and transference of Ti alloy to the Zr [19]. At lower fretting frequencies (1 Hz), less delimited shallower wear scars indicated reduced surface damage. At 2 Hz fretting frequency, there was a heavily damaged central zone with an external area of spread material. There were multiple clear and dark striations indicative of corrosion pits. At 4 Hz, a concise and delimited wear scar was observed with severe material degradation at the central part. Multiple striations suggestive of a predominant abrasive behaviour at all frequencies were seen. At lower fretting frequencies, the wear scar pattern was mainly due to the micro-fragmentation of fine oxide particles. As the frequency increased (4 Hz), fracture and particle detachment occurred, forming a deeper wear track, predominantly in the central part. Deficient passive film increased the counter body penetration with continuous surface damage at higher fretting frequencies. At lower frequencies, the micro-fragmented oxide particles were observed, while full metallic particles were predominant at higher frequencies [20].

The EDS analysis showed pure Ti with some Al, C, Si, F, Ca, K, and Cl depending upon the composition of the electrolyte and metal transfer during fretting corrosion [16, 17, 21]. It was suggested that the oxide layer's chemical composition determined the material's mechanical properties. The native oxide layer on CpTi4 is composed mainly of TiO_2_ and Al_2_O_3_ and V_2_O_3_ in the case of Ti6Al4V. The ZrO_2_ and Nb_2_O_5_ were also present onTi13Nb13Zr. Different oxides in the protective layer induce defects and reduce their cohesive and adhesive properties. It facilitates the removal of the remaining passive film during the sliding. In the presence of most aggressive conditions, i.e., artificial saliva with pH3.5 and1000 ppm F, the EDS revealed dark areas composed of Ti, O, and F, suggestive of fluoride incorporation into the debris or the surface, resulting in a less protective film [9]. The machined and acid-etched samples revealed the presence of Ti, C, and Si. The Si probably resulted from polishing with colloidal silica, while the sandblasted disks showed the presence of Al due to sandblasting with Al_2_O_3_ particles [17]. Precipitation of calcium fluoride on CpTi4 was also seen in EDS [16]. The analysis of the worn surfaces showed elements from the salts in the artificial saliva and sulphur in the presence of proteins. At the periphery of the wear scar, higher levels of elements from the accumulated debris were observed [25]. The EDS data in various studies also reported the transfer of particles like Zr and Al on the surface of Ti during fretting corrosion against different materials like Roxolid or Ti6Al4V alloy [18, 19]. This material transfer from Ti6Al4V to Ti during fretting corrosion was called *“fretting stir welding”* [21].

### 3.2. Factors Influencing Bio-Tribocorrosion of Ti Dental Implants in Oral Cavity

The included studies evaluated numerous factors that may cause bio-tribocorrosion of dental implants in the oral cavity (Table 1) (Figure 2(b)). They are as follows.

#### 3.2.1. Type of Ti Alloys

The Ti exists in two forms, the alpha phase at room temperature and the beta phase at temperatures above 833°C [18]. The alpha structure has more surface oxides leading to superior biocompatibility, while the beta phase with less surface oxides has more strength. The CpTi (grades 1–4) primarily comprises alpha phase grains making it more biocompatible while Ti6Al4V alloy (grade 5) is a dual alpha/beta phase alloy with added vanadium which stabilizes the more extensive beta phase. As the Ti6Al4V alloy has less abundant surface oxides, it is more susceptible to corrosion [18]. Moreover, it is hard and brittle, increasing its tribocorrosion susceptibility [19].

In the current review, the tribocorrosion was reported in the dental implants made of CpTi [8–10, 12, 16, 17, 21, 24], Ti6Al4V [8, 11, 13–15, 18–25], NiCr [22–24], Ti13Nb13Zr [9], Zr [19, 21, 25], and Roxolid [18]. Of the various grades, grade 2 [10, 16, 17, 24], grade 4 [9, 21], and grade 5 [8, 11, 13–15, 18–25] were evaluated in the included studies.

The TiZr alloy (Roxolid) has a binary alpha structure as Zr has identical allotropic transformation and phase transition temperature to those of Ti. It has a smaller grain size with increased surface oxides which improves its corrosion resistance compared to the Ti6Al4V [18]. Other alloys like Ti13Nb13Zr have a higher wear rate than CpTi and reduced re-passivation [9]. The NiCr alloy has higher corrosion resistance than both CpTi and Ti6Al4V alloy [22–24]. Studies also revealed more significant wear-accelerated corrosion of the sintered materials than the cast alloys, as a more passive layer was incorporated in the tribolayer of the sintered material [13].

#### 3.2.2. Oral Biofilm

The degradation of the metal surface, when exposed to the metabolic products from microorganisms in a biofilm, causes microbial corrosion [41]. The main types of bacteria associated with the corrosion of metals are sulphate-reducing bacteria, sulphur-oxidizing bacteria, iron oxidizers, iron reducers, manganese oxidizers, and microbes. They secrete organic acids and extracellular polymeric substances, which degrade the metal surface [41].

The saliva and its constituents in the oral cavity are an excellent environment for biofilms, especially at the micro gaps on the dental implant interfaces [3]. The pathogens accumulate and penetrate up to about 10 µm gaps at the implant interfaces. They induce pitting corrosion which deteriorates the implant material [42]. It is induced by two mechanisms: (a) Metal dissolution by organic acids produced during sugar catabolism, which reduces the pH of the oral environment and (b) Deposition of a biofilm on the dental implant, which creates a differential oxygen potential on the surface [43].

In-vitro studies revealed that lower pH and increased immersion time in saliva, along with mechanical movement and contact with dissimilar metals, enhanced the metal dissolution process [43–45]. The differential oxygen levels on the surface of Ti produced less aerated zones, which acted as anodes. They underwent crevice corrosion and released metal ions into the saliva. Together with the bacterial end products and chloride ions, they promoted metal degradation [43].

Both early (e.g., *S. mutans, Streptococccus gordonii* (*S. gordonii*)*, and Lactobacilli*) and late (e.g., *P. gingivalis*) colonizers and the bridging organisms (e.g., *Fusobacterium nucleatum* (*F. nucleatum*)) of the oral biofilm cause corrosion [16, 46–48]. The early pathogens produce lactic acid, hydrogen peroxide (H_2_O_2_), and formic acid, decreasing pH [49]. The lactic acid causes pitting and surface discoloration. The late colonizers produce LPS that degrade the TiO_2_ and increase the tissue inflammatory response [46]. They change the oxidation state of Ti and corrode its surface in the absence of mechanical loads.

Besides, the commensal fungus *Candida albicans* (*C. albicans*), which co-aggregates with *S. mutans*, has been isolated from the peri-implant areas [16]. The biofilms of *S. mutans* and *C. albicans,* when cultured on the ground and polished CpTi plates, showed increased biomass production with roughness. The reciprocating sliding tests on both the surfaces, with and without biofilms, in the presence or absence of fluoride in artificial saliva, showed that the biofilms had a lubricating effect and significantly influenced the tribological properties [16].

The microbial adhesion and succeeding biofilm formation are enhanced by surface roughness as it increases the colonization area [16, 49]. As the roughness threshold that influences bacterial plaque accumulation is 0.2 *μ*m, any surface with more roughness is highly susceptible to microbial accumulation [42, 50]. Accordingly, there was increased adhesion and proliferation of *S. sanguis* on the rough acid-etched surfaces of Ti dental implant coated with hydroxyapatite (HA). Its corrosion was enhanced by van der Waals, electrostatic interactions, and hydrodynamic forces [42].

Contrarily, exposure of Ti6Al4V alloy to the electrolyte solution containing *S. gordonii* with or without *F. nucleatum* showed a reduced corrosion rate due to the formation of a passive TiO_2_ film by these bacteria. However, at 96 hours, the electrochemical potential of the solution of *F. nucleatum* with *S. gordonii* was altered with an increased tendency towards corrosion [47]. The *F. nucleatum* and *Prevotella melaninogenica* are Gram-negative anaerobic sulphate-producing pathogens that produce butyric acid, carbon dioxide, and hydrogen during the enzymatic degradation of saccharides and increase the Ti corrosion. Its corrosion rate is proportional to the *S. gordonii* levels [47].

An analysis of five failed Ti dental implants showed that biofilm formation caused severe pitting and scratching of the smooth collars of the implant [43]. The surface discoloration was related to the acidic environment and inflammatory reactions. There was a permanent breakdown of the oxide film, releasing the metal ions and debris in vivo and hindering the reintegration of the implant [43].

It was also observed that fluorides and biofilms together had a lubricating effect on Ti during sliding motion [16]. The biofilm protected the TiO_2_ layer by undergoing a plastic deformation during sliding. The ruptured and agglomerated exopolymeric matrix rolled along the sliding track and protected the Ti substrate. The fluoride formed calcium fluoride (a solid lubricant), which reduced Ti's friction, wear, and corrosion [16]. After several sliding cycles, when the biofilm thickness decreases, there is greater diffusion of fluoride ions through the extracellular matrix. They reach the micro-canals inside the biofilms and disrupt and detach them from the sliding track. Therefore, fluorides generally seen in oral mouth rinses can significantly affect the bio-tribocorrosion behaviour of Ti in the oral cavity [16].

Besides, LPS degraded CpTi and Ti6Al4V alloy under sliding test conditions. It was suggested that before sliding, the polysaccharide part of LPS attacked the oxide film, producing some defects and exposing the Ti. The remaining passive film was removed during the sliding, and the LPS attacked the new Ti surface. Although a new passive film was formed after the sliding stopped, it was less protective than the native film resulting in corrosion [8]. Studies suggested that the LPS accelerate Ti's wear/corrosion process [8, 16]. The presence of oral infections like periodontitis may cause significant biofilm accumulation and lower the implant prognosis [8].

#### 3.2.3. Low pH and Fluorides

The acidic pH of saliva due to dietary, therapeutic, or bacterial metabolism plays a significant role in the bio-tribocorrosion of dental implants [48]. Any inflammation or infections like periodontitis or systemic conditions reduce the pH of the surrounding environment. The lactic, hydrochloric, and hydrofluoric (HF) acids are mainly involved in corrosion [48]. The HF acid is derived from the fluoride ions present in oral hygiene products like acidic fluoridated toothpaste, mouthwashes, or cariostatic gels [48, 51, 52]. The chemical reactivity of Ti with HF acid depends on the exposure time and concentration. Usually, it is resistant to corrosion in artificial saliva at low or nil fluoride ion concentration and at a pH of 7.5 [53]. This is due to surface passivation caused by TiO_2_, Al_2_O_3_, and V_2_O_3_. However, increased immersion time in higher fluoride concentration interferes with the formation of the passive layer. The TiO_2_ reacts with fluoride to form a soluble Ti-F complex, including Na_2_TiF_6_, TiCl_6_, and TiF_6_. They increase porosity and decrease its corrosion resistance [51].

Dental implants are exposed to various changes in pH, temperature, and saliva, leading to chemical corrosion during mastication [12]. The influence of salivary fluoride and pH variations was evaluated on the tribocorrosion behaviour of CpTi, Ti6Al4V, Ti13Nb13Zr, and NiCr alloys [9, 13, 14, 22, 24]. In general, the pH of the electrolyte ranged between 3 and 8 [8–10, 12, 14, 16, 18–20, 22, 24, 25] and fluoride concentration between 0 and 1000 ppm [9, 13, 14, 16, 22]. The near-*β*Ti13Nb13Zr alloy and the CpTi4 became less passivating when immersed in a solution containing 1000 ppm of fluoride at a pH of 3.5 [9].

During rubbing, a sudden drop in the potential towards cathodic values indicated mechanical depassivation. The low pH and high fluoride concentration hindered passivating film formation on the metal surface, leading to corrosion [9]. The tribocorrosion behaviour of cast and sintered Ti6Al4V biomedical alloy was tested in artificial human saliva at three different pH values (3, 6, and 9) and only in acidic saliva with 1000 ppm fluorides; both cast and sintered Ti alloys showed tribocorrosion independent of the pH. The addition of fluorides to the acidified solution caused active Ti alloy dissolution [14]. Similarly, the most significant fluctuation and weight loss of the CpTi was observed at the pH of 6.0 when subjected to sliding tests. It was suggested that the CpTi might undergo degradation at near-neutral pH in the presence of motion. At pH 6.0, the protective passive film layer is not reformed cohesively, resulting in more tribocorrosion products at the surface, which are easily sheared off. Therefore, as the average pH of the oral cavity is 6.3, it may increase the risk for dental implant degradation [12].

At 0, 190, 570, and 1140 ppm of fluoride ions in artificial saliva, Ti6Al4V alloy showed a cathodic shift in the fretting corrosion potential due to the damage to the passive film. Instant re-passivation was observed in artificial saliva without fluorides after the cessation of the fretting motion. It was suggested that the fluorides hinder instantaneous re-passivation of the damaged areas and the wear volume of the fretted zone increased with the increasing fluoride ion concentration [11].

The studies showed that fluorides (20 to 12300 ppm) and 35% H_2_O_2_ at low pH were detrimental to Ti and its alloys (e.g., Ti6Al4V and Ti13Nb13Zr) [9, 15, 23, 51, 52, 54, 55]. The mouthwashes containing amine stannous fluoride and chlorhexidine (0.2%) promoted localized corrosion [56]. The chlorhexidine gluconate (0.01%) corroded Ti implant surfaces when rubbed for long periods [17, 57, 58]. Comparing different treatments used to detoxify dental implants revealed that immersion or rubbing of implants with these solutions at pH < 3 enhanced corrosion. They did not corrode the surface at neutral to basic pH [59]. It was found that acidic rubbing treatments were more aggressive than immersion because rubbing resulted in little or no oxide layer re-passivation as mechanical forces were continuously applied [44, 59]. Moreover, strong acids such as peroxyacetic and citric acid dissolved the oxide layer to a greater degree under abrasion than with immersion, suggestive of their tribocorrosive effect [59] Contrarily, fluoride at 227 ppm and low pH may act as a lubricating agent during sliding due to the formation of calcium fluoride (CaF2) or fluorohydroxyapatite. The CaF_2_ is a solid lubricant in tribology that reduces friction and wear. It prevents adhesion, enables tribo-chemical reactions, and lowers the shear strength [16]. Some studies have suggested surface homogenization of CpTi and Ti6Al4V alloy upon prolonged contact with fluorides in dentifrices, mouthwashes, and neutral pH. There was reduced adherence of *S. mutans* with no significant surface degradation [60].

Considering the adverse effects of fluorides on Ti at low pH, they should be avoided in patients with dental implants. Besides, crevice-free implants or the application of HF acid-resistant coatings should be considered [61].

#### 3.2.4. Galvanic Interactions

The galvanic interactions between NiCr and Ti6Al4V alloys were evaluated for both less (227 ppm, pH 5.5) and more aggressive (12,300 ppm, pH 4.0) combinations of fluoride and pH [22]. The Ti6Al4V presented a decreasing corrosion resistance with increasing fluoride concentration and decreasing pH. The more aggressive solution resulted in higher Ti volume loss regardless of its coupling with NiCr. The higher fluoride concentration reduced tribocorrosion resistance of Ti6Al4V. However, when coupled with NiCr, Ti6Al4V was able to achieve passivity and did not exhibit adverse galvanic effects with the different fluoride combinations tested [22, 24]. The improvement in the Ti6Al4V corrosion resistance in fluoride, when coupled with NiCr, indicates the safe use of fluorides for Ti6Al4V dental implants associated with NiCr-based prostheses and implant connections [23].

Like NiCr, couplings of Ti with Zr and Roxolid were evaluated. The Ti/Ti groups had the highest voltage drop indicating greater corrosion susceptibility, while the Zr/Roxolid group had the lowest voltage drop and minimal electrochemical degradation. The Ti/Ti group had the most significant wear volume loss, while the Zr/Ti group had the least. There was about 5 to 6 times more wear of Ti than their Zr counterparts, with Zr/Ti group being the best and Ti/Ti being the worst coupling [18, 21].

#### 3.2.5. Mastication Frequencies

In the oral cavity, the dental implants are exposed to cyclic occlusal loading during masticatory activity leading to the mobility of their joint components [20]. These micro-motions remove the TiO_2_ layer and affect the implant-bone interface and internal components like the abutment, screws, and crowns [20, 62, 63]. This process of accelerated surface damage at the interface of contacting materials subjected to low amplitude oscillatory movements is called fretting [64]. The ingress and egress of saliva accelerate it in between implant and alloy superstructure, creating areas of differential oxygen potential, which enhance corrosion [48, 56]. In the areas of low oxygen concentration, the surface becomes anodic. As corrosion is directly proportional to the ratio between cathode and anode, the dissolution is hastened when the anode is of low dimensions [56].

The fretting motions influence the depassivation–repassivation processes on Ti6Al4V alloy surface. The mechanical motion in a simulated oral environment at frequencies 1, 2, and 4 Hz degraded the TiO_2_ layer on Ti6Al4V discs. The lower fretting frequencies enabled the re-passivation of Ti6Al4V and produced a protective barrier against degradation [20]. In the presence of parafunctional habits, multidirectional occlusal forces of various intensities and frequencies act on dental implants and disrupt the TiO_2_ layer. In laboratory testing, a frequency of 4 Hz may hinder its re-passivation [20].

#### 3.2.6. Saliva

The lubricants in the saliva affect the tribocorrosion process. For instance, when the ball-on-plate tests evaluated the tribological behaviour of the ZrO_2_/Ti6Al4V pair in dry and lubricated conditions, the Ti plate always presented a higher coefficient of friction than the Ti ball. It was suggested that the degradation and regeneration processes of the Ti passivating film differed in the two configurations during sliding.

The saliva contains several organic compounds like amino acids (e.g., leucine, glycine, glutamate, and aspartate), proteins (e.g., albumin, statherin, and histatin), and glycoproteins (e.g., mucin) that play a significant role during tribocorrosion. Among the various lubricants, human saliva produced the lowest coefficient of friction and minor wear [19]. The addition of albumin, urea, lysozyme, and mucin to artificial saliva during triboactivity testing of the Ti6Al4V/Zr pair showed that albumin and mucin adsorbed more on the surface. Although tribocorrosion was present in all the systems, it was lowest in the presence of mucin [25]. The salivary mucin forms a physically crosslinked network that promotes a viscoelastic effect on the Ti and counteracts the sliding surfaces [10, 25]. It reduces the saliva viscosity and CoF values. Besides, proteins like albumin adsorb on the prosthetic materials leading to reduced wear and friction. Under fretting, Ti's tribocorrosion behaviour is slightly improved after adding the citric acid or anodic inhibitor to artificial saliva due to oxidation and reduction reactions occurring in the contact area [10].

### 3.3. Toxic Implications of Ti Bio-Tribocorrosion

Ti ions released from dental implants due to bio-tribocorrosion may accumulate in the local tissues. Its levels should not exceed 15 mg per 70 kg body weight in a healthy individual [65]. Its levels last from a few hours to several months as it is highly insoluble and difficult to be eliminated from the body [38, 65, 66]. The particles are accumulated in the surrounding tissues of the dental implant [65, 67]. They are released at a prolonged rate without any systemic immune response, and their effects are often unnoticeable. However, the excessive concentration of Ti particles destroys the oral intraepithelial homeostasis, promotes peri-implant tissue inflammation, and affects the osteoblasts and osteoclasts with subsequent bone loss around the implants [65]. Furthermore, they enter into systemic circulation via the bloodstream, accumulate in the distal organs, and cause allergies and hyper-sensitivity reactions [65, 66] (Table 2) (Figure 2(b)). The following section discusses these effects of Ti particles.

#### 3.3.1. Cytotoxic Effects

The cytotoxic effects of Ti particles depend on their size and concentration in the surrounding tissues. The submicron and micron-sized particles trigger a pro-inflammatory response and are engulfed by the inflammatory cells [48]. At high concentrations, they induce necrosis of the gingival epithelial cells. At about 5 ppm, they significantly increased CCL2 mRNA expression in gingival epithelial cells exposed to LPS derived from *P. gingivalis*. Moreover, the mRNA expression levels of TLR-4 and ICAM-1 were significantly increased in gingival epithelial cells loaded with Ti ions at 9 ppm. Therefore, the Ti ions increased the sensitivity of gingival epithelial cells to microorganisms and promoted monocyte infiltration in the oral cavity leading to cytotoxicity and inflammation at the implant-gingival tissue interface [27].

The 1 to10 µm diameter Ti ions released from the dental implant biofilm removal were cytotoxic to fibroblasts [39]. A comparative evaluation of cytotoxic effects of CpTi and its alloy Ti6Al4V on human gingival fibroblast showed better cell viability with CpTi. The aluminum and vanadium in the Ti6Al4V alloy caused cytotoxicity [32]. The mucosa adjacent to Ti screws showed increased macrophages and T lymphocytes infiltration, resulting in an immune response [28].

Furthermore, increased numbers of macrophages and T lymphocytes associated with Ti particles were observed in the human mucosal biopsies. They were found inside and outside the epithelial cells and macrophages in the peri-implant mucosa. Although their concentration was higher in the peri-implantitis group, the particles were released irrespective of inflammation [5]. They were toxic to the peri-implant cells like osteoblasts, fibroblasts, and lymphocytes. Besides, CD68+ cells associated with particle engulfed monocytes were observed at these sites [38].

Elevated levels of Ti ions cause osteoblast toxicity in adjacent bone tissues and degrade the prognosis of implant survival. The Hippo/YAP signalling pathway is involved in Ti ions–induced osteoblast toxicity. Ti ions (10 ppm) inhibit osteoblastic growth and differentiation by inducing nuclear expression of YAP in them [35].

#### 3.3.2. Increased Proinflammatory Cytokines

As the Ti ions and the degraded oxide particles act as foreign debris, they induce chronic peri-implant inflammation. In vitro studies revealed that activated macrophages secreted pro-inflammatory cytokines (e.g., interleukin [IL]-1*β*, IL-6, TNF-*α*, and RANKL), chemokines, growth factors, prostanoids, and degradative enzymes [33, 48] which further promoted bone and soft tissue destruction, clinically visible as peri-implant mucositis or peri-implantitis [31].

#### 3.3.3. Peri-Implantitis and Bone Loss

Ti-induced cytotoxicity and inflammation accelerate bone loss in the peri-implant region. Its dissolution modifies the peri-implant microbiome structure and diversity [34]. The biofilm formation at supracrevicular or intracrevicular implant surfaces is dependent on the surface roughness, surface energy, and hardness. The Gram-negative periodontopathogens like *P. gingivalis* produce LPS, promoting inflammation in the peri-implant environment. The LPS increase Ti corrosion and tribocorrosion, further accelerating its adherence to Ti surface at acidic pH [29]. Besides, the defects in the oxide layer enhance this process due to surface energy changes or chemical modification. As LPS exhibit low surface energy, they are attracted to sites with increased roughness and higher surface energies, as seen on corroded Ti surfaces.

Moreover, they exhibit greater adherence on Ti6Al4V alloy surface than the CpTi due to the following: (a) Increased saturation of CpTi surface with LPS at high concentrations, (b) the difference in oxide film composition and surface energy of CpTi and Ti6Al4V alloy, and (c) greater surface energy of Ti6Al4V alloy due to its higher surface hardness [29]. However, greater attachment of *S. mutans* and *P. gingivalis* was observed on corroded CpTi when compared to Ti6AlV alloy due to differences in their physicochemical and antimicrobial properties [26, 30].

Ti particles trigger foreign body reactions, and their severity depends on the quantity and physicochemical properties of the metallic particles and the host response. The multinucleated giant cells and osteoclasts generated by the fusion of macrophages in the peri-implant region promote the osteolytic process. In addition, mixed pro-inflammatory cytokines like RANKL, IL-33, and TGF-*β*1 are increased in the presence of Ti particles. Since RANKL stimulates osteoclastic bone resorption and reduces the apoptosis of osteoclasts, more bone loss occurs in the areas with Ti particles. Besides, higher levels of TGF-*β*1 inhibit osteoblastic proliferation and mineralization and oppose the actions of bone morphogenic proteins, thereby hindering bone formation. The higher levels of IL-33 indicated increased cell signalling related to peri-implantitis [38, 68]. In addition, the presence of Ti ions in submucosal plaque around implants also supported the association between Ti and peri-implantitis [31]. They inhibited HA crystal growth causing local osteolysis and hindered osseointegration.

#### 3.3.4. Hypersensitivity and Allergy

Literature has revealed that patients sensitive to Ti may develop pruritus, redness, swelling, and skin eczema. Facial eczema has been reported in patients receiving Ti dental implants. A cross-sectional observational study on 270 subjects visiting a dental metal allergy clinic reported that about 6.3% of patients were allergic to Ti. The main symptom was eczema in a patient with Ti dental implant, and the reaction ceased after its removal [37]. A case of multiple cutaneous fistulae was reported after the placement of dental implants. It was suggested that loose Ti particles resulting from corrosion could activate response pathways to DNA damage in oral epithelial cells. The activation of these pathways caused homeostatic imbalance leading to epithelial barrier violation and, a more significant infiltration of the immune response, development of complications like fistulae. Besides, type IV hypersensitivity reactions to Ti (100–300 ppm) may cause gingival enlargement, mucosal hyperaemia, facial eczema, and rash [40]. They may combine with endogenous proteins to form antigenic molecules as they have a high affinity for proteins. These antigenic molecules are captured by Langerhans cells and cause a delayed-type of hypersensitivity reaction with repeated contact [69]. Besides, Ti particles have been associated with glottis edema and spontaneous exfoliation of implants [70].

#### 3.3.5. Genotoxicity

Ti in various forms may lead to site-specific epigenetic modification, as they stimulate a strong immune response. The site-specific methylation of genes leads to peri-implantitis. A case-control study evaluated global DNA methylation patterns in cases of healthy implants and peri-implantitis and its association with Ti dissolution [37].

#### 3.3.6. Other Toxic Reactions

The Ti particles released into the soft tissues may cause metallosis [62]. They may disseminate to other body organs like lungs, kidneys, and liver. Although they do not have a genotoxic effect on osteoblasts and fibroblasts, they have been associated with neoplasias like squamous cell carcinoma, osteosarcoma, and plasmacytoma of the mandible [70]. Furthermore, TiO_2_ is categorized as a likely carcinogen to humans (Group 2B of carcinogens) by the International Agency for Research on Cancer (IARC), although the actual carcinogenic potential of dental implants is still questionable [70].

### 3.4. Methods to Reduce the Bio-Tribocorrosion of Dental Implants

As the tribocorrosion is dependent on the physical, chemical, mechanical, and structural properties of dental implant materials, various methods have been applied to improve their tribocorrosion resistance. Newer alloys of Ti, including *β* and near *β* Ti alloys like Ti13Nb13Zr and Ti5Zr, were investigated for reducing the tribocorrosion. While Ti5Zr exhibits optimal tribocorrosion and surface features, Ti13Nb13Zr had similar or slightly inferior tribocorrosive properties to CpTi [9].

Various surface modification techniques including porous Ti [14, 71], plasma electrolytic oxidation (PEO) [72], anodizing [1, 73, 74], nitriding [75], micro arc oxidation [1, 76], poly-ether-ether-ketone (PEEK) [77], and biofunctionalization using the peptides and Ti coatings doped with Ca, P, Si, Ag [76, 78, 79], and Mg [1] were evaluated to improve the tribocorrosion resistance of Ti dental implants.

The porous Ti alloys for implants are beneficial as porous structure mimics the natural bone, allowing the bone to grow into the pores which results in a better fixation of the artificial implant [14]. Nano structuring by ultrasonic shot peening is an annealing treatment that produces a more prominent surface nanostructure with an increased number, density, and sharper grain boundaries. It reduces the corrosion rate by 86.2% due to more effective surface passivation [80]. The micro-arc oxidation yields bio-functional oxide films resistant to tribocorrosion [76]. Similarly, the oxygen plasma immersion ion implantation treatments enhanced the corrosion resistance and cell adhesion of the Ti surface due to the increased surface thickness of TiO_2_ [81].

The CpTi disks polished and coated with TiN and silicon carbide exhibited lower corrosion [82]. The TiN film coated on Ti-Nb alloys containing Nb up to 40 wt% showed increased pitting corrosion resistance than the alloys with less percentage of Nb [83]. A crystalline cubic zirconia (ZrO_2_) nanocoating on CpTi demonstrated good biocompatibility and corrosion resistance [84]. Similarly, coating with either HA or partially stabilized zirconia (PSZ) or a mixture of 50 percent HA and PSZ increased corrosion resistance of CpTi and Ti6Al4V alloys [85]. CpTi was also coated with HA alone or a mixture of strontium, HA, and TiO_2_ to reduce surface corrosion [86].

The graphene coating on CpTi and Ti6Al4V also increased the corrosion resistance. This coating increased the resistance to mechanical stresses and electrochemical stability and reduced the tendency for surface oxidation and degradation [87]. The higher Ca/P ratios and the addition of Ag nanoparticles into the oxide layer improved the surface properties, tribocorrosive behaviour, and cell responses of CpTi [88]. Similarly, Ag and copper nanoparticle coatings on dental implant screws inhibited the production of sulphur, chlorine, and sodium [89]. Moreover, veneering PEEK to Ti6Al4V reduced the wear rate and coefficient of friction [77].

Other coatings like tantalum nitride (TaN) exhibited higher resistance to micro-biocorrosion when compared to bare Ti and TiN coating in vitro. The TaN-decorated Ti possessed increased antibacterial resistance with increased integrity and stability [90]. The physical vapor deposition of either TiN or ZrN on the CpTi significantly reduced the number of adherent bacteria and, hence, micro-biocorrosion [91]. The TiN surfaces have shown similar results in other studies as well [92].

The zinc-decorated Ti surfaces exhibited excellent corrosion resistance when exposed to excessive H_2_O_2_. They inhibited the adhesion and proliferation of macrophages and promoted healing and tissue reconstruction. They improved the oxidative microenvironment around the materials by increasing the expressions of antioxidant enzyme relative genes in macrophages. Subsequently, they provided excellent corrosion resistance and osseointegration capacity [93].

Some newer deposition methods like PEO have been applied to synthesize bioactive glass-based coating (PEO-BG) on Ti materials. The PEO-BG coated Ti had superior mechanical and tribological properties with higher corrosion resistance. They reduced the pathogenic bacterial biofilms and promoted adsorption of blood plasma proteins without cytotoxic effects on human cells [94].

The studies included in the review suggest that tribocorrosion degrades the Ti dental implants and releases Ti ions that plausibly lead to peri-implantitis. However, these results should be treated with caution as they are based on conclusions obtained from in-vitro experimental studies. Besides, multiple factors may cause tribocorrosion of Ti dental implants in the oral cavity. Clinical trials involving failed Ti dental implants should be done to further verify these results.

## 4. Conclusions

The published results in the review show that Ti, although a biocompatible and mechanically stable dental implant material, is not inert to degradation. It is highly susceptible to bio-tribocorrosion in a hostile oral environment. The oral biofilms combined with low pH, fluorides, and masticatory activity affect the implant surface. Low pH due to dietary factors or infections and increased fluoride exposure from dentifrices may promote Ti dissolution. Subsequently, the metal particles released due to corrosion increase peri-implant tissue inflammation, hyper-sensitivity, and allergic reactions. Even though methods to reduce Ti degradation in the oral cavity are essential, any structural modifications may affect its corrosion resistance. Further research investigating the corrosion of Ti in an oro-systemic environment and methods to control it are warranted.

## Figures and Tables

**Table 1:** Studies evaluating the factors responsible for bio-tribocorrosion of titanium dental implants.

Authors	Type of Implant material	Factor evaluated
Vieira et al. 2006 [10]	CpTi grade 2	(i) Fretting (ii) pH
Sivakumar et al. 2011 [11]	Ti6Al4V alloy	(i) Fluoride
Mathew et al. 2012 [8]	CpTi Ti6Al4V alloy	(i) Biofilm
Mathew et al. 2012 [12]	CpTi	(i) pH
Licausi et al. 2013 [13]	Ti6Al4V alloy	(i) Fluoride
Licausi et al. 2013 [14]	Ti6Al4V alloy	(i) pH
Faverani et al. 2014 [15]	Ti6Al4V	(i) Mouthwash
Cruz et al. 2015 [16]	CpTi	(i) Fluoride (ii) Biofilm
Golvano et al. 2015 [9]	CpTi *β*-Ti13Nb13Zr	(i) fluoride (ii) pH
Beline et al. 2016 [17]	CpTi (grade 2)	(i) Mouthwash
Sikora et al. 2018 [18]	Ti6Al4V alloy TiZr (Roxolid)	(i) Metal couplings
Branco et al. 2019 [19]	ZrO_2_ Ti6Al4V	(i) Lubricants
Alfaro et al. 2019 [20]	Ti6Al4V	(i) Fretting frequencies
Corne et al. 2019 [21]	CpTi (grade 4) Ti6Al4V	(i) Metal couplings
Barros et al. 2020 [22]	Ti6Al4V NiCr	(i) Galvanic interactions (ii) Fluoride (iii) pH
Dos Reis Barros et al. 2020 [23]	Ti6Al4V NiCr	(i) Galvanic interactions
Barros et al. 2021 [24]	NiCr CpTi Ti6Al4V	(i) Galvanic interactions (ii) pH
Teixeria et al. 2021 [25]	Ti6Al4V/Zr pair	(i) Albumin (ii) Urea (iii) Lysozyme (iv) Mucin

**Table 2:** Various toxic effects of titanium particles.

Authors	Type of study	Influence on oral cavity
Correa et al. 2009 [26]	In vitro	(i) Greater *S. mutans* adherence
Makihira et al. 2010 [27]	In vitro	(i) Increased cytotoxicity and inflammation
Olmedo et al. 2012 [28]	Ex-vivo	(i) Increased number of macrophages and T lymphocytes cause bone loss and implant failure
Barao et al. 2013 [29]	In vitro	(i) Increased LPS adherence
Olmedo et al. 2013 [5]	Ex-vivo	(i) Metal particles exfoliated in peri-implant mucosa trigger inflammatory reactions
Barao et al. 2014 [30]	In vitro	(i) Increased *P. gingivalis* attachment on implant surface
Safioti et al. 2017 [31]	Observational study	(i) Peri-implantitis
Chandar et al. 2017 [32]	In vitro	(i) Greater cell viability of CpTi than Ti6Al4V (ii) Aluminum and vanadium in Ti6Al4V induce cytotoxicity (iii) Cytotoxicity decreases due to the formation of TiO2
Pettersson et al. 2017 [33]	In vitro	(i) Increased Ti ions near Ti implants stimulate human macrophages to release IL-1*β* specifically from LPS stimulated macrophages
Daubert et al. 2018 [34]	Observational study	(i) Ti ions modify peri-implant microbiome structure and diversity (ii) Increased Peri-implantitis
Zhu et al. 2018 [35]	In vitro	(i) Ti ions (10 ppm) suppressed osteoblasts and trigger nuclear expression of YAP pathway (ii) Its activation suppresses osteogenic differentiation
Hosoki et al. 2018 [36]	Observational study	(i) Ti allergy occurred in 6.3% of all cases
Daubert et al. 2019 [37]	Case control study	(i) Increased methylated DNA cytosine in peri-implantitis due to epigenetic alterations in the tissues (ii) Increased association between Ti concentration and global methylation levels independent of peri-implantitis (iii) Methylation of DNA influenced by Ti dissolution
Berryman et al. 2020 [38]	Ex-vivo	(i) Increased bone loss and peri-implantitis
Kotsakis et al. 2020 [39]	In vitro	(i) Cytotoxic effects of Ti on fibroblasts
De Lima-Souza et al. 2021 [40]	Case report	(i) Erythematous-papular-nodular lesions in mandibular and submandibular region (ii) Histopathologically chronic fistula with foreign body reaction and Ti ions along fistula wall

## Data Availability

All the data used to support the findings of this review are included within the article.
